# Differences between first and fourth year medical students’ interest in pursuing careers in academic medicine

**DOI:** 10.5116/ijme.571b.af3d

**Published:** 2016-05-24

**Authors:** Monica H. Vetter, Mary Carter

**Affiliations:** 1 Medical School, University of Louisville, Louisville, KY, USA

**Keywords:** Medical students, careers, academia

## Abstract

**Objectives:**

The purpose of this pilot study was to assess the differences in the attitudes of first and fourth-year medical students regarding careers in academics. We also sought to identify any factors associated with an increased interest in academic medicine.

**Methods:**

A cross-sectional study was conducted during October 2013 at the University of Louisville.  All first and fourth year medical students were invited to complete an online survey utilizing a survey instrument developed through literature review. Demographic data and information about background experiences were collected in addition to participants' perceptions regarding careers in academia using a 5-point Likert scale. Participants were also queried about their current interest in a career in academics and the likelihood they would pursue academic medicine.

**Results:**

Of the 330 potential participants, 140 (42.4%) agreed to participate. Overall, fourth-years reported a higher likelihood of pursuing an academic career than first-years. Research experience, publications, distinction track interest or involvement, and belief that a career in academics would reduce salary potential were positively correlated with reported likelihood of pursuing academic medicine.

**Conclusions:**

Findings from this pilot study demonstrate differences in interest in academic medicine between junior and senior medical students. Additionally, several factors were associated with a high likelihood of self-reported interest in academic. Based on these findings, efforts to increase medical students’ interest in academic medicine careers could be supported by providing more research and teaching opportunities or distinction track options as a structured part of the medical school curriculum.

## Introduction

In March 2015, a report was released at the request of the Association of American Medical Colleges (AAMC) detailing a projected shortage of physicians in the United States by 2025.^1^ This study forecasts that by 2025, the demand for physicians will exceed supply by 46,000 to 90,000.  In response to these projected physician shortages, there has been a growth in medical student enrollment. First-year medical student enrollment for the 2016-2017 academic year is expected to reach 21,376, representing a 29.6% increase from first-year enrollment in 2002-2003.^2^ Given this increase in medical school enrollment, there is an increasing need for academic physicians and preceptors. Additionally, academic health centers are being asked to expand their capacity for care to address the growing health care needs of Americans.^3^

Further issues affecting the supply of academic physicians include difficulties with retention and recruitment. Per a 2008 AAMC report, only 52% of all faculty included in a study remained at their medical school after a 10 year period.^4^ Furthermore, 38% of faculty left academics altogether. Much of the reported discontent of medical school faculty members appears to be related to problems balancing career and family and a lack of career development and support. In one study, 42% of medical school faculty reported they were “seriously considering leaving academic medicine in the next five years”.^5^ Finally, expected retirement of tenured faculty as well as an increased desire for part-time schedules by physicians may also impact the overall supply of academicians.^6^   The combined challenges of physician recruitment and retention in careers in academic medicine represent a critical issue in medical education.

Much of the research on factors influential on academic career choices has centered on resident and attending physicians. Interest in a career in academic medicine has been found to be positively correlated with several factors including the completion of a graduate degree or fellowship and publishing research during training.^7^ Having a role model or mentor is also suggested to be an important influence in choosing academic medicine.^6^ Additionally, the literature outlines numerous obstacles to developing academic physicians such as a loss of interest in academics during residency^8^ as well as concerns about salary and debts.^6^ Interestingly, factors influencing the likelihood of a physician entering into academic medicine appears to differ based on gender and specialty choices.^9^

Despite the above research, there have been very few studies that explore medical students’ attitudes towards academic medicine careers.  One recent study of third- and fourth-year medical students identified the following factors as predictors of a student's self-reported desire for academic medicine: interest in teaching, research administration, and community service opportunities.^10^  There was also a positive correlation between medical school debt and interest in academic medicine.

At this time, it is unknown if there are any differences between junior and senior medical students' perceptions of academic medicine. Therefore, the purpose of this study was twofold: 1) To ascertain if there were any differences in reported interest in or perceptions of academic medicine between newly enrolled and upper classmen medical students, and 2) Determination of what factors were associated with an increased interest in academic medicine in a medical student population. It was hypothesized that a higher proportion of fourth-year medical students would report a desire to pursue academic medicine compared with naïve first-year students. Factors that would be positively correlated with a higher reported likelihood of pursuing a career in academics were hypothesized to be the following: previous teaching experience, identification of a mentor, having an advanced degree, reported interest in a distinction track during medical school and structured extracurricular research experience. 

## Methods

### Study design

This was a cross-sectional study conducted during the academic year 2012-2013 at the University of Louisville. The study was declared exempt by the University of Louisville’s Institutional Review Board.  A 27-item survey was administered to participate via electronic survey. Participants were sent three reminder emails over a six to eight week period to ensure an optimal response rate.

### Participants

All first and fourth year medical students enrolled in the 2012-2013 academic year at the University of Louisville School of Medicine were invited to participate in this study resulting in 330 potential participants. Participants were invited to participate via email utilizing the University of Louisville School of Medicine list serve maintained by the Medical School Student Affairs Office. Completion of the survey represented participant consent.

### Survey design and instrument

A 27-item online survey was developed based on literature review and factors previously identify as influential on future career choice. Following creation, the survey was then reviewed for content and format by several professional medical educators. This data included demographic items, quantification of research and teaching experience, presence of a mentor in academics, and reported interest in in a distinction track or medical education electives. Participants were asked to both answer yes/no to “Considering an Academic Medicine Career” and to rate the current likelihood that they will pursue academic medicine as a career after residency on a scale from 0-100 percent. Lastly, perceptions of agreement to positively worded stems were measured using a 5-point Likert scale anchored with strongly disagree (1) and strongly agree (5).  Opinions assessed included appeal of research and teaching both during and after residency, desire to be a role model, and the effects of a career in academic medicine on patient contact and potential annual salary.  Finally, students were invited to report any thoughts or concerns about pursuing a career in academics in a free response item.

### Data collection

Respondents completed all surveys online and were downloaded into an anonymous spreadsheet. The data were stored on a secure server.

### Data analysis

Data were analyzed utilizing SPSS Version 21.0.  Categorical data such as demographics were compared using Chi square.  Normally distributed continuous variables were compared with independent t-tests.  Spearman correlates were used to analyze data on prior teaching and research experience and the likelihood of pursuing a career in academic medicine.  The critical *p* value was set at < 0.05.

## Results

### Demographics

Of the 330 medical students invited to participate, 140 responses were recorded giving a response rate of 42.4%. There were 62 first-year and 78 fourth-year participants. Chi squared analyses were performed showing no significant differences of gender (χ^2^_(1) _= 1.402, *p* = .236), race (χ^2^_(1) _= .004, *p* = .947), or possession of advance degrees (χ^2^_(1) _= 3.270, *p* = .071) between the first-year and fourth-year cohorts. Independent T-tests demonstrated no significant differences between the cohorts’ response rate or research experience (see Table 1). As expected, fourth-year participants were significantly older than their first-year counterparts. Fourth-year participants also reported significantly more teaching experience when compared to first-years.

**Table 1:** Comparison of parametric demographic data of the first- and fourth-year cohorts

Variable		M	SD	t	df	p	d
Age				-6.76	135	<.001	1.13
	First-year	24.0	2.24				
	Fourth-year	26.4	2.00				
Teaching experience				-2.28	127.7	.025	.4
	First-year	9.68	10.87				
	Fourth-year	14.78	14.66				
Research experience				-1.03	129	.303	.18
	First-year	15.19	17.13				
	Fourth-year	18.62	20.0				

### Likelihood of pursuing a career in academic medicine

In response to a yes/no question asking whether participants were considering a career in academic medicine, fourth-years were significantly more likely to report consideration of academic medicine (χ^2^_(1) _= 8.145, *p* = .004).  Fourth-years also ranked the likelihood of pursuing academic medicine (*M* = 54.4,* SD* = 29.4) significantly higher on a scale of 0-100 than their first-year counterparts (*M *= 42.1, *SD *= 29.3), *t*_(__126)_ = -2.358, *p* = .020. Pearson correlation coefficients were calculated for the self-reported likelihood of pursuing a career in academics with participants’ background information (see Table 2).  The self-reported likelihood of pursuing a career in academic medicine was positively correlated with the following: interest in a distinction track, total number of co-authored papers/posters/presentations, report of a significant relationship with a mentor, research experience, and teaching experience. Possession of an advanced degree was not found to be correlated with the self-reported likelihood of pursuing a career in academic medicine.

**Table 2:** Pearson correlation between likelihood of pursuing an academic medicine career and demographic variables

Variable	r	p
Interest in distinction tract	0.35	.001
Total number of co-authored papers/posters	0.24	.006
Had a significant relationship with a mentor	0.21	.016
Had an advanced degree	0.20	.175
Research experience	0.20	.026
Teaching experience	0.18	.049

### Likert-Scale Responses

Independent T-tests were performed to compare Likert-scale responses between cohorts regarding attitudes and beliefs about future career goals (see Table 3). Fourth year participants more strongly agreed with the following statements than first years:  1) I want to be involved in research during residency, 2) Academics would increase opportunities for research, 3) I want to be involved with teaching during/after residency, and 4) I want to be a role model. Although fourth-years indicated more interested in a career in academic medicine, they were also more likely to indicate agreement with the belief that an academic career would reduce salary potential. Pearson correlation demonstrated a weak correlation between belief of reduction of salary potential with academic medicine and participant’s reported consideration of academic medicine (*r* = .24, *p* = .007) and the scaled likelihood of pursuing a career in academics (*r* = .20, *p* = .022).

**Table 3:** Likert-scale responses by year in medical school year

Statement	M	SD	t	df	p	d
I want to be involved in research during residency.			-2.76	126	.007	.5
First-year	3.2	1.07				
Fourth-year	3.75	1.2				
Academic medicine would increase my opportunities to do research.			-2.64	126	.009	.2
First-year	3.84	0.95				
Fourth-year	4.26	0.84				
I want to be involved in teaching during residency.			-5.61	126	<.001	1.0
First-year	3.7	1.06				
Fourth-year	4.58	0.73				
I want to be involved in teaching after residency.			-3.03	126	.003	.5
First-year	3.84	1.12				
Fourth-year	4.35	0.77				
I want to be a role model for future medical students, residents, and physicians.			-3.17	126	.002	.6
First-year	4.30	0.71				
Fourth-year	4.65	0.53				
Academic medicine would reduce salary potential.			-3.56	125	.001	0.6
First-year	3.47	0.74				
Fourth-year	3.98	0.88				

## Discussion

Our study investigated the differences between junior and senior medical students and their reported interest in academic medicine. In this study, fourth-years had a greater self-reported likelihood of pursuing a career in academic medicine than first-year participants. We also explored what factors were associated with the participants’ self-reported likelihood of pursuing career.  Our results indicated the anticipated likelihood of pursuing a career in academics was positively correlated with the following factors: expressed interest in a distinction track, number of publications, relationship with a mentor, research experience, and teaching experience.  These results are concordant with other previously identified factors noted to play a role in the decision to pursue a career track as an academic physician.^6^^,^^10^ Based on these data, medical schools could potentially support medical student interest in academic medicine by providing distinction track options, structured research opportunities and increased teaching opportunities during the 4-year curriculum. We also identified mentorship as an associated factor so we recommend consideration of formal mentorships with academic physicians starting in the early years of medical student education

There are potentially numerous reasons why fourth-years were more likely to report a desire for a career in academic medicine than first-years.  One of which may be an increased awareness of the variety of career opportunities as the trainee progresses through medical school. Fourth-year participants also had more interactions with academic physicians than first-years as expected, which may have increased the opportunities to form relationships with an academic mentor.  Finally, it was also possible that fourth-year participants had increased idealism about the future as they approach graduation and are therefore more likely to overlook perceived negatives about careers in academic medicine.

Unexpectedly, there was a positive correlation between the perception that academic medicine lowered salary potential and the reported likelihood of entering academic medicine. We hypothesize that as students proceeded through medical school they realized other benefits of working in academic medicine such as increased research support and more opportunities to mentor young physicians and medical students.  As salary and debt concerns have been identified in numerous studies^3^^,^^10^ as factors contributing to interest in academic medicine, future research and educational policy changes in this area are warranted.

Our study had several limitations. First, this pilot study was a single institution, cross-sectional study, and our results may not apply outside the borders of our institution. Therefore, further studies from multiple institutions is warranted. Additionally, this study included only medical students and is potentially not applicable to the resident population. Finally, our response rate was 42%. However, other studies in which medical students were surveyed indicated an average response rate of only 50%.^11^ Further steps could include a multi-institutional study to provide a more representative sample of the medical student population as well as a prospective cohort study, which would allow for the monitoring of how interest in academics changes throughout the course of medical school and residency.

## Conclusions

Our study illustrates differences between junior and senior medical students’ interest level in academic medicine. We have also identified several factors that are positively correlated with interest in a career as academic medicine. Given the predicted shortage of physicians, increasing medical school enrollment, and issues with retention of academic physicians, understanding what influences medical students’ career choices can shed light on ways medical educators can foster the interest in careers in academic medicine. This, in turn, could help secure the pipeline of future academic physicians.

### Conflict of Interest

The authors declare that they have no conflict of interest.
